# Rapid Degradation of Phenanthrene by Using *Sphingomonas* sp. GY2B Immobilized in Calcium Alginate Gel Beads

**DOI:** 10.3390/ijerph6092470

**Published:** 2009-09-16

**Authors:** Xue-Qin Tao, Gui-Ning Lu, Jie-Ping Liu, Ting Li, Li-Ni Yang

**Affiliations:** 1 School of Environmental Science and Engineering, Zhongkai University of Agriculture and Engineering, Guangzhou 510225, China; E-Mails:ljpzhk@126.com (J.-P.L.);litzhku@163.com (T.L.);lnyang@yeah.net (L.-N.Y.); 2 School of Chemistry and Chemical Engineering, South China University of Technology, Guangzhou 510640, China; E-Mail:lgn36@qq.com

**Keywords:** artificial seawater, immobilization, phenanthrene biodegradation, polycyclic aromatic hydrocarbons (PAHs), *Sphingomonas* sp. GY2B

## Abstract

The strain *Sphingomonas* sp. GY2B is a high efficient phenanthrene-degrading strain isolated from crude oil contaminated soils that displays a broad-spectrum degradation ability towards PAHs and related aromatic compounds. This paper reports embedding immobilization of strain GY2B in calcium alginate gel beads and the rapid degradation of phenanthrene by the embedded strains. Results showed that embedded immobilized strains had high degradation percentages both in mineral salts medium (MSM) and 80% artificial seawater (AS) media, and had higher phenanthrene degradation efficiency than the free strains. More than 90% phenanthrene (100 mg·L^−1^) was degraded within 36 h, and the phenanthrene degradation percentages were >99.8% after 72 h for immobilized strains. 80% AS had significant negative effect on the phenanthrene degradation rate (PDR) of strain GY2B during the linear-decreasing stage of incubation and preadsorption of cells onto rice straw could improve the PDR of embedded strain GY2B. The immobilization of strain GY2B possesses a good potential for application in the treatment of industrial wastewater containing phenanthrene and other related aromatic compounds.

## 1. Introduction

Phenanthrene is hazardous to aquatic life, plants and many other organisms and has often been used as a representative carcinogenic polycyclic aromatic hydrocarbon (PAH) containing both “bay-region” and “K-region” [1]. Effective degradation of phenanthrene is necessary in order to preserve the environment and human health and efficient degradation methods are available [2–6]. Compared to physico-chemical methods, biodegradation is regarded as an interesting alternative method to detoxify or remove phenanthrene from the environment because of lower costs and the possibility of complete mineralization [7,8]. In this sense, biodegradation of phenanthrene has therefore attracted increasing attention and a large number of phenanthrene-degrading bacteria and fungi, such as *Sphingomonas* [9], *Pseudomonas* [10], *Mycobacterium* [11], *Nocardioides* [12], *Burkholderia* [13], *Staphylococcus* [14], *Sinorhizobium* [15] and *Penicillium* [16] species, have been isolated and characterized at the physiological and genetic level.

*Sphingomonas* sp. was shown to have unique genes for degradation of phenanthrene and other PAHs [17]. The gene is distantly related in sequence homology and genorganization to those of *Pseudomonas* and other genera reported so far [18]. Strain *Sphingomonas* sp. GY2B was shown to efficiently use phenanthrene as the sole carbon and energy source in mineral salts medium and artificial seawater [19,20]. In addition to phenanthrene, strain GY2B could use a broad range of PAHs and related aromatic compounds such as naphthalene, 2-naphthol, salicylic acid, catechol, phenol, benzene and toluene as a sole source of carbon and energy [21].

The use of free microorganisms for bioremediation of contaminated sites can fail because the inoculants must be able to overcome biotic and abiotic stresses in the environment in which they are introduced, and might cause other problems such as secondary pollution by the inoculants [22]. Immobilization of microorganisms offers several advantages over free cells, including protecting cells from the toxic effects of hazardous compounds and increasing their survival and metabolic activity in bioremediation systems [23], and therefore has been receiving increasing attention [24–26].

Calcium-alginate cross-linking is one of the most commonly used immobilization methods because the procedure is simple, relatively mild and does not have any toxic effects on the cells [27–29]. The purpose of this work is to study the phenanthrene degradation efficiency of *Sphingomonas* sp. GY2B cells immobilized in calcium alginate gel beads, and to explore the environmental adaptability and potential application of the strain.

## 2. Materials and Methods

### 2.1. Materials and Growth Conditions

Phenanthrene-degrading strain *Sphingomonas* sp. GY2B was isolated from crude oil contaminated soils collected at a site near Guangzhou Petrifaction Company, China [19]. Phenanthrene was purchased from Aldrich Company (USA, purity above 98%) and its hexane stock solution at 10 g·L^−1^ was prepared and stored in brown bottle placed at 4 °C. All other chemicals used were of the highest purity available.

The phenanthrene-degrading strain was routinely growth at 30 °C in mineral salts medium (MSM) consisiting of following (per liter): 5 mL phosphate buffer solution (KH_2_PO_4_, 8.5 g·L^−1^; K_2_HPO_4_·H_2_O, 21.75 g·L^−1^; Na_2_HPO_4_·12H_2_O, 33.4 g·L^−1^; (NH_4_)Cl, 5.0 g·L^−1^); 3.0 mL MgSO_4_ solution (22.5 g·L^−1^); 1.0 mL FeCl_3_ solution (0.25 g·L^−1^); 1.0 mL CaCl_2_ solution (36.4 g·L^−1^); 1.0 mL trace element solution (MnSO_4_·H_2_O, 39.9 mg·L^−1^; ZnSO_4_·H_2_O, 42.8 mg·L^−1^; (NH_4_)_6_Mo_7_O_24_·4H_2_O, 34.7 mg·L^−1^). Its pH was adjusted to 7.2–7.4 with 5 mol·L^−1^ HCl and NaOH solutions.

A previous study had shown that the addition of 85% artificial seawater (AS) had very low impact on the growth and phenanthrene degradation ability of strain *Sphingomonas* sp. GY2B [20]. The AS composition was as follows (per liter) [30]: 24.5 g NaCl; 0.03 g H_3_BO_3_; 1.54 g CaCl_2_·2H_2_O; 4.09 g Na_2_SO_4_; 0.1 g KBr; 0.003 g NaF; 0.2 g NaHCO_3_; 0.7 g KCl; 0.017 g SrCl_2_·6H_2_O; 11.1 g MgCl_2_·6H_2_O. Its pH was adjusted to 7.5 with 5 mol·L^−1^ HCl and NaOH solutions. In this study, 80% AS (MSM:AS = 20:80, v/v) was prepared for phenanthrene degradation experiment.

Nutrient agar was consisted of 10 g·L^−1^ peptone, 5 g·L^−1^ beef extract, 5 g·L^−1^ NaCl, and 2.0% agar. All the media and solutions were prepared with distillated water and autoclaved at 1 atm for 15 min. All experiments were carried out in 100 mg·L^−1^ phenanthrene.

### 2.2. Cultivation of Microorganisms

Rice straw was used as adsorption carrier and obtained from local farmers in Guangzhou, China. Before any pre-treatment, rice straw was washed thoroughly with tap water until the washings were clean and colourless and then it was ground into powder after air drying.

First, 0.5 mL hexane stock solution of phenanthrene was added to sterilized flasks, forming a thin film of phenanthrene on the flask bottom after evaporation of hexane, then 45 mL sterilized MSM was added. The flasks with 45 mL MSM were inoculated with 1.5 g rice straw powder and 5 mL cell culture of strain GY2B activated in MSM with 100 mg·L^−1^ phenanthrene for 2 d, and then were incubated at 30 °C on a shake preset at 150 rpm under dark condition. In addition, activated strain GY2B was spread on nutrient agar plates and placed in a artificial climate incubator preset at 30 °C at the same time.

After 2 d, the strain GY2B adsorbed and grew on the rice straw at the late exponential phase, was harvested with gauze and rinsed with 0.90% normal saline twice, and then was kept in 10 mL normal saline at 4 °C. The colonies of strain GY2B grew on the nutrient agar plates for 2 d were also picked off and kept in 10 mL normal saline at 4 °C to use as the cell culture without preadsorption.

### 2.3. Cell immobilization Method

Different concentration (2%, 3% and 4%, w/v) of sodium alginate solutions were prepared and sterilized. Then 30 mL sodium alginate solution was mixed with 10 mL of normal saline containing cell culture to form sodium alginate-cell suspensions. The sodium alginate-cell suspensions were added dropwise to well-stirred, sterilized 2% (w/v) CaCl_2_ solutions using a syringe without pinhead. Calcium-alginate gel beads having a spherical shape (about 2 mm in radius) were formed instantly. Beads were left in CaCl_2_ solution with gentle stirring for 3~4 h to allow them to harden. They were then harvested with gauze and rinsed with 0.90% normal saline. Before use for degradation studies, bead surfaces were blotted dry with paper towels. Because of the instantaneous formation of cross-linked alginate on the surface of the droplets, all cells were assumed to be trapped within the beads and no measurements were taken to determine if any cell loss occurred.

### 2.4. Mass Transfer Performance and Mechanical Stability Tests

Three harvested beads were added into flask with 200 mL distillated water, and then two drops of methylene blue solution were dropped into the flask to investigate the mass transfer performance by observing the infiltration speed of methylene blue into the beads. About ten beads were added into flask with 50 mL normal saline and then were incubated at 30 °C on a shake preset at 150 rpm for 2 d to see whether the beads would break up to qualitatively describe the mechanical stability. In addition, the smoothness and resiliency of the beads were qualitatively described by pinching them with finger.

### 2.5. Biodegradation Test with Free and Immobilized Strains

The biodegradation of 100 mg·L^−1^ phenanthrene in 50 mL (including inoculant culture) MSM or 80% AS were investigated by inoculating 5 mL normal saline cell suspension or gel beads prepared from equal amount of cell suspensions. The flasks were incubated at 30 °C and 150 rpm. Flask samples were taken at 6–24 h intervals over 4 d to detect the residual phenanthrene in the flask. Flasks having no inoculant were set up as control test. All assays were carried out in triplicate.

### 2.6. Analysis Methods

Quantitative analysis of phenanthrene in the flask was performed by extracting the whole flask cultures with 20 mL cyclohexane twice. The two extracts were combined and dried with anhydrous Na_2_SO_4_. The solvents were diluted with cyclohexane to certain volumes (or after evaporated under reduced pressure). The detection of phenanthrene in cyclohexane solution was performed using UVVis spectroscopy (UV752N) by measuring the absorbance at 254 nm (OD_254_) [3]. The linearity range of this method in 0.04~4.0 mg·L^−1^ is quite good because the correlation coefficient between the phenanthrene concentration and OD_254_ was always >0.999. Control tests with varied phenanthrene concentrations were run to assess the recovery efficiency under extraction condition, which was >85% at phenanthrene concentration as low as 0.01 mg·L^−1^.

## 3. Results and Discussion

### 3.1. Effect of Concentration of Na-Alginate Solution on the Beads

Different concentration of Na-alginate solutions were investigated to get Ca-alginate gel beads performance and stability, and the qualitative results are listed in Table 1.

As shown in Table 1, a low concentration (2%) of embedding carrier couldn’t form good spherical beads but rather formed unregular ellipsoidal and clubbed gels, while regular white spherical beads were formed when the concentrations of sodium alginate were 3% and 4%. The mechanical stability increased with the increase of sodium alginate concentration, however, the mass transfer performance decreased in the same time. Though 4% of sodium alginate possessed four excellent results, the important pollutant transfer index was bad. Considering the mass transfer performance and mechanical stability demands of bioremediation of contaminated sites, 3% sodium alginate was selected for the subsequent experiments.

### 3.2. Phenanthrene Degradation by Embedded and Free Cells

**Figure 1.**
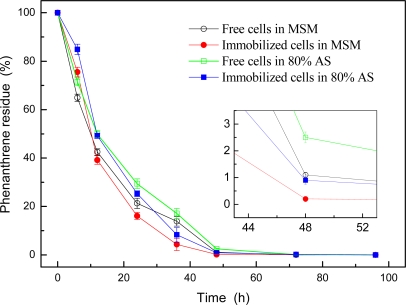
Phenanthrene degradation trends of free and immobilized strain GY2B. The abiotic loss of phenanthrene in control test in 96 h was 15.1%.

The phenanthrene degradation trends of free and immobilized strain GY2B in MSM and 80% AS media were shown in Figure 1. Results showed the immobilized strain has high degradation percentage in both media, and has higher phenanthrene degradation efficiency than the free strain in its exponential growth phase.

As can be seen in Figure 1, more than 97.5% phenanthrene was degraded at the first 48 h. This effect was especially observed within the first 12 h where about 50% phenanthrene was degraded in both free and immobilized cells and either in MSM or in 80% AS. In addition, >99.8% phenanthrene was degraded within 72 h and was completely degraded in 96 h. The phenanthrene degradation percentages of immobilized strain were slightly lower than that of the free strain at the first 6 h of incubation in both media. However, the trends were reversed after this period that the immobilized strain had larger phenanthrene degradation percentages than that of the free strain from 6 h to the end of degradation. The reversion of trends indicated that the immobilized cells could increase the stability of the microbial system and hold high phenanthrene degradation activity longer than the free cells, and further proved the advantages of immobilization.

### 3.3. Phenanthrene Degradation by Embedded Cells Preadsorbed on Rice Straw

**Figure 2.**
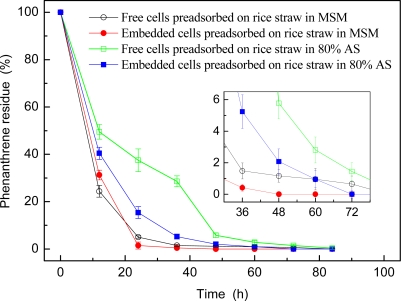
Phenanthrene degradation trends of strain GY2B preadsorbed on rice straw. The abiotic loss of phenanthrene in control test in 84 h was 12.4%.

The phenanthrene degradation trends of free and embedded strain GY2B preadsorbed on rice straw in MSM and 80% AS media are shown in Figure 2. As can be seen in the figure, phenanthrene was rapidly degraded in the first 48 h, especially that >50% phenanthrene was degraded within the first 12 h for both free and embedded cells either in MSM or in 80% AS. More than 98.5% phenanthrene was degraded in MSM within 36 h and here AS has obvious effect on the phenanthrene degradation efficiency of free cells preadsorbed on rice straw. Phenanthrene was completely degraded within 84 h except the free cells in 80% AS was 99.6%.

Comparing with Figure 1, the preadsorption of strain GY2B on rice straw could obviously enhance the phenanthrene degradation efficiency in MSM for both embedded and unembedded cells, and also could slightly enhance the phenanthrene degradation efficiency in 80% AS for embedded cells. These results indicated the embedding immobilization of strain GY2B with preadsorption on rice straw can enhance the phenanthrene degradation efficiency of the strain.

### 3.4. Phenanthrene Degradation Rates

For better comparison of the biodegradation efficiency by immobilized strain GY2B with the free strain, the degradation data were further reduced to obtain the phenanthrene degradation rate (PDR) during the linear-decreasing stage of incubation with the following equation (1) [10,14,21] and listed in Table 2.

(1) $$\text{PDR}\;=\;\frac{c(0)\;-\;c(t)}{t}$$

where *c* is the concentration of phenanthrene and *t* is the rapid linear-decreasing degradation period.

The calculated results of PDR clearly demonstracted the negative effect of AS and the enhancement of immobilization on the phenanthrene degradation efficiency of strain GY2B during the linear-decreasing stage of incubation. Embedding immobilization of cells in calcium alginate gel beads could obviously increase PDR of the strain and preadsorption of cells on rice straw could increase PDR of embedded strain GY2B.

The PDR of free strain GY2B in a previous study in the same condition was 3.45 mg·L^−1^·h^−1^ [21], slightly larger than 3.28 mg·L^−1^·h^−1^ in the present study. Tian *et al.* [10] reported the PDR of a free strain of *Pseudomonas mendocina* who could degrade 95.0% of 100 mg·L^−1^ phenanthrene in 2 d was 2.61 mg·L^−1^·h^−1^. Mallick and Dutta [14] reported the PDR of *Staphylococcus* sp. strain PN/Y at initial concentration of 100 mg·L^−1^ phenanthrene was 0.89 mg·L^−1^·h^−1^, significantly less than that of GY2B in the same initial concentration.

### 3.5. Potential Application of Strain GY2B

Phenanthrene and its metabolites are distributed either as natural or artificial aromatic compounds in various environmental sites. Strain GY2B had high phenanthrene degradation efficiency, however, the use of free bacterial cells for wastewater treatment in activated sludge processes creates problems such as solid waste disposal [24]. Immobilized microorganisms have been shown to be effective for treating wastewater with little sludge production [31,32]. Hence, immobilization of strain GY2B possesses a good application potential in the treatment of industrial wastewater containing phenanthrene and other related aromatic compounds.

Studies have shown that modified rice straw was a good biosorbent for some pollutants in water, such as oil-spills [33], dyes [34] and heavy metal ions [35]. In this study, embedded strain GY2B preadsorbed on raw rice straw showed higher phenanthrene degradation efficiency than the free strain and direct embedded strain, which might be a result of the following: (1) rice straw contains many different carbon sources so the adsorption of cells onto the straw probably lead to an increase in cell numbers and (2) the adsorption of phenanthrene onto the rice straw promotes the degradation efficiency of the strain adsorbed on it. Modification of rice straw might enhance the adsorption of phenanthrene onto the carrier and further improve the phenanthrene degradation efficiency of strain GY2B, further investigation on this issue is in progress.

## 4. Conclusions

Embedding immobilization of strain *Sphingomonas* sp. GY2B in calcium alginate gel beads was investigated and rapid phenanthrene degradation was detected on the embedded strains. Results showed that the embedding immobilized strain had high degradation percentages in both mineral salts medium (MSM) and 80% artificial seawater (AS) media, and had higher phenanthrene degradation efficiency than the free strain. 80% AS had significant negative effect on the phenanthrene degradation rate (PDR) of strain GY2B during the linear-decreasing stage of incubation and preadsorption of cells onto rice straw could improve the PDR of embedded strain GY2B. Immobilization of strain GY2B possesses a good application potential in the treatment of wastewater containing phenanthrene and other related aromatic compounds.

## Figures and Tables

**Table 1.** Effect of concentration of Na-alginate solution on the beads.

**Na-alginate concentration**	**2%**	**3%**	**4%**
Spherical molding characteristic	Bad	Good	Excellent
Mechanical stability	Bad	Good	Excellent
Mass transfer performance	Excellent	Good	Bad
Smoothness	Bad	Good	Excellent
Resiliency	Bad	Good	Excellent

**Table 2.** Phenanthrene degradation rates (PDR) of free and immobilized strain GY2B during the linear-decreasing stage of incubation ^a^.

**No.**	**Inoculants**	**Preadsorbed on rice straw**	**Media**	**Incubation period (h)**^b^	***c*(0) (mg·L^−1^)**	***c*(*t*) (mg·L^−1^)**	**PDR (mg·L^−1^·h^−1^)**
1	Free	No	MSM	0~24	100 a	21.3 a	3.28 ± 0.09 a
2	Immobilized	No	MSM	0~24	100 a	16.1 b	3.50 ± 0.07 b
3	Free	No	80% AS	0~24	100 a	29.4 c	2.94 ± 0.09 c
4	Immobilized	No	80% AS	0~24	100 a	25.3 d	3.11 ± 0.05 a
5	Free	Yes	MSM	0~24	100 a	5.03 e	3.96 ± 0.02 d
6	Immobilized	Yes	MSM	0~24	100 a	1.47 f	4.11 ± 0.02 e
7	Free	Yes	80% AS	0~24	100 a	37.5 g	2.60 ± 0.16 f
8	Immobilized	Yes	80% AS	0~24	100 a	15.4 b	3.53 ± 0.11 b

a. Means followed by the same letter within each column are not significantly different at the 0.05 level according to one-way ANOVA test.

b. The linear-decreasing time interval involved in the calculation of PDR.
